# Regional Brain Recovery from Acute Synaptic Injury in Simian Immunodeficiency Virus-Infected Rhesus Macaques Associates with Heme Oxygenase Isoform Expression

**DOI:** 10.1128/JVI.01102-20

**Published:** 2020-09-15

**Authors:** Yoelvis Garcia-Mesa, Rolando Garza, Maria E. Diaz Ortiz, Analise L. Gruenewald, Brandon L. Bastien, Rebecca Lobrovich, David J. Irwin, Michael R. Betts, Guido Silvestri, Dennis L. Kolson

**Affiliations:** a Department of Neurology, Perelman School of Medicine, University of Pennsylvania, Philadelphia, Pennsylvania, USA; b Department of Genetics, Perelman School of Medicine, University of Pennsylvania, Philadelphia, Pennsylvania, USA; c Department of Microbiology, Perelman School of Medicine, University of Pennsylvania, Philadelphia, Pennsylvania, USA; d Department of Medicine, Division of Infectious Diseases, Emory University School of Medicine, Druid Hills, Georgia, USA; Ulm University Medical Center

**Keywords:** HO-1, HO-2, brain, brainstem, heme oxygenase, human immunodeficiency virus, neuroinflammation, oxidative stress, simian immunodeficiency virus

## Abstract

Brain injury occurs within days in simian immunodeficiency virus (SIV) or human immunodeficiency virus (HIV) infection, and some recovery may occur within weeks. Inflammation and oxidative stress associate with such injury, but what drives recovery is unknown. Chronic HIV infection associates with reduced brain frontal cortex expression of the antioxidant/anti-inflammatory enzyme heme oxygenase-1 (HO-1) and increased neuroinflammation in individuals with cognitive impairment. We hypothesized that acute regional brain injury and recovery associate with differences in regional brain HO-1 expression. Using SIV-infected rhesus macaques, we analyzed multiple brain regions through acute and chronic infection (90 days postinfection [dpi]) and quantified viral (SIV *gag* RNA), synaptic (PSD-95; synaptophysin), axonal (neurofilament/neurofilament light chain [NFL]), inflammatory, and antioxidant (enzymes, including heme oxygenase isoforms [HO-1, HO-2]) markers. PSD-95 was reduced in the brainstem, basal ganglia, neocortex, and cerebellum within 13 dpi, indicating acute synaptic injury throughout the brain. All areas except the brainstem recovered. Unchanged NFL was consistent with no acute axonal injury. SIV RNA expression was highest in the brainstem throughout infection, and it associated with neuroinflammation. Surprisingly, during the synaptic injury and recovery phases, HO-2, and not HO-1, progressively decreased in the brainstem. Thus, acute SIV synaptic injury occurs throughout the brain, with spontaneous recovery in regions other than the brainstem. Within the brainstem, the high SIV load and inflammation, along with reduction of HO-2, may impair recovery. In other brain regions, stable HO-2 expression, with or without increasing HO-1, may promote recovery. Our data support roles for heme oxygenase isoforms in modulating recovery from synaptic injury in SIV infection and suggest their therapeutic targeting for promoting neuronal recovery.

**IMPORTANCE** Brain injury induced by acute simian (or human) immunodeficiency virus infection may persist or spontaneously resolve in different brain regions. Identifying the host factor(s) that promotes spontaneous recovery from such injury may reveal targets for therapeutic drug strategies for promoting recovery from acute neuronal injury. The gradual recovery from such injury observed in many, but not all, brain regions in the rhesus macaque model is consistent with the possible existence of a therapeutic window of opportunity for intervening to promote recovery, even in those regions not showing spontaneous recovery. In persons living with human immunodeficiency virus infection, such neuroprotective treatments could ultimately be considered as adjuncts to the initiation of antiretroviral drug therapy.

## Abstract (precis)

Brain injury induced by acute simian (or human) immunodeficiency virus infection may persist or spontaneously resolve in different brain regions. Identifying the host factor(s) that promotes spontaneous recovery from such injury may reveal targets for therapeutic drug strategies for promoting recovery from acute neuronal injury. The gradual recovery from such injury observed in many, but not all, brain regions in the rhesus macaque model is consistent with the possible existence of a therapeutic window of opportunity for intervening to promote recovery, even in those regions not showing spontaneous recovery. In persons living with human immunodeficiency virus infection, such neuroprotective treatments could ultimately be considered as adjuncts to the initiation of antiretroviral drug therapy.

## INTRODUCTION

Acute central nervous system (CNS) infection with simian immunodeficiency virus (SIV) or human immunodeficiency virus (HIV) is associated with neuroinflammation, neuronal injury, and metabolic changes, each of which has unclear long-term significance (1). In untreated SIV infection, acute brain injury (frontal cortex) may spontaneously resolve within weeks, depending on the infecting SIV strain used and the immunological status of the host animal. However, chronic, untreated SIV infection often leads to severe neurodegeneration associated with neuronal death and encephalitis (1, 2). The host factors that determine regional brain vulnerability to, and the potential for recovery from, acute neuronal injury due to SIV or HIV infection are unknown.

Natural history studies of acute SIV infection that apply brain neuroimaging combined with postmortem pathological analysis have yielded various results. These studies (typically ≤14 days post-SIV infection, with peak viremia at 11 to 12 days postinfection [dpi]) have differed with respect to the infecting SIV strain used and whether the animals are experimentally immunosuppressed by CD8^+^ T lymphocyte depletion (anti-CD8 monoclonal antibody infusions) to enhance virus replication and accelerate CNS pathogenesis (1, 3–6). In a study using SIV_mac251_ (SIV swarm containing both T lymphocyte- and macrophage-tropic SIV strains), neuroimaging evidence for transient neuronal injury (*N*-acetylaspartate [NAA] loss) and glial inflammation (increased choline/creatinine) in the frontal cortex was observed (14 dpi) (7). Another SIV_mac251_ study demonstrated acute inflammation without neuronal injury in the basal ganglia through 4 weeks postinfection (8). Notably, the frontal cortex neuronal injury appeared to resolve within several weeks, as evidenced by increasing NAA levels (8). Subsequent immunohistological studies of the frontal cortex in a subset of these macaques confirmed reduced synaptophysin (presynaptic marker) staining (14 dpi), but no reduction in neuronal cell counts, which was consistent with the neuroimaging data indicating reversible neuronal injury (9). In particular, these macaque studies were performed without inducing immunosuppression through CD8^+^ T lymphocyte depletion to accelerate neuropathogenesis.

In contrast, more-severe SIV-induced neuronal injury and “accelerated” neuropathogenesis is observed in animals immunosuppressed with CD8^+^ T lymphocyte depletion (10). Such T lymphocyte depletion after SIV_mac251_ inoculation can result in progressive irreversible neuronal injury and death in the frontal cortex and basal ganglia over 8 weeks (11). A comprehensive analysis of this accelerated SIV neuropathogenesis model revealed (i) progressive and irreversible frontal cortex neuronal injury (synaptophysin and NAA loss), (ii) an association between frontal cortex SIV RNA levels and neuronal injury, and (iii) attenuation of progressive neuronal injury with the use of antiretroviral drugs to suppress SIV replication (5). In this model, there is no evidence for recovery from neuronal injury, although a limited neuroprotective effect of host CD8^+^ T lymphocyte responses, presumably through control of SIV replication, is suggested. Other endogenous host-protective responses have yet to be defined.

We therefore sought to determine whether acute SIV_mac251_ infection of rhesus macaques results in transient or persistent injury throughout the brain, and whether specific host responses (inflammatory, antioxidant) associate with acute neuronal injury and recovery. We examined (i) the brainstem (medulla, pons, midbrain), which serves not only motor functions but also critical autonomic functions; (ii) the neocortex (parietal cortex, frontal cortex, prefrontal cortex, deep frontal lobe), involved in higher neurocognitive functions; (iii) the basal ganglia, which serve primitive motor functions; and (iv) the cerebellum, which modulates motor coordination. Each anatomic region, with the exception of the cerebellum, is commonly affected in SIV and HIV infection. We also examined specific neuronal markers of particular relevance to SIV/HIV neuropathogenesis, which can alter neuronal integrity (synapses, axons). Among these are synaptic markers (PSD-95, synaptophysin, and Homer-1) and an axonal marker (neurofilament light chain [NFL]), as well as inflammatory markers (interferon [IFN] types I and II) and glial fibrillary acidic protein (GFAP) (astrocytes). We also quantified the protein expression of antioxidant response enzymes [NAD(P)H quinone oxidoreductase 1 (NQO1), glutathione peroxidase 1 (GPX1), peroxiredoxin 1 (Prdx1), and heme oxygenase isoforms (HO-1, HO-2)].

We focused on host antioxidant response genes, which execute cytoprotective responses to acute cellular injury. Among those linked to neuronal injury and recovery is heme oxygenase (HO), an antioxidant/anti-inflammatory enzyme (12). The HO-1 isoform is robustly inducible by multiple triggers, while HO-2 is considered to be constitutively expressed and modestly inducible by a few factors. Each has been implicated in neuroprotective responses in brain injury models (13–17).

Our previous studies demonstrated that reduced HO-1 expression is associated with neuroinflammation and cognitive impairment in people living with HIV (PLWH) (18) and with the release of excitotoxic levels of glutamate from HIV-infected macrophages (19). In this macaque study, we observed specific regional brain differences in SIV RNA levels, inflammatory markers, and antioxidant enzyme expression that associated with synaptic integrity. The expression patterns show that low expression of HO isoforms occurs in the presence of high SIV RNA levels and high neuroinflammation, concurrently with reduced recovery from acute synaptic injury in the brainstem. Expression of HO-1 was lower in the brainstem and cerebellum than in the basal ganglia and neocortex, and expression of HO-2 in the brainstem was lower than that in the cerebellum and similar to that in the basal ganglia and neocortex. These general patterns of HO-1 and HO-2 expression did not clearly associate with vulnerability to acute synaptic injury, which was observed throughout the brain. However, recovery from acute synaptic injury associated with HO isoform expression. HO-2 expression decreased progressively in the brainstem, where the SIV load and neuroinflammation were highest, and where synaptic recovery was not observed. HO-2 remained stable elsewhere, where recovery was observed. HO-1 expression did not decrease in any region, and it increased in the neocortex during the synaptic recovery phase.

## RESULTS

### SIV establishes infection throughout the brainstem, basal ganglia, neocortex, and cerebellum in rhesus macaques within 10 days postinoculation.

**FIG 1:**
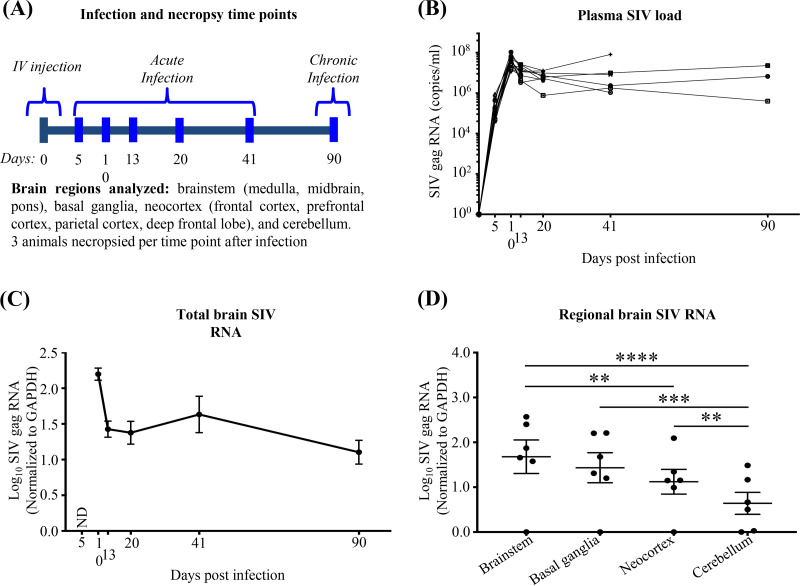
SIV RNA is detectable in plasma and brain tissue through 90 dpi. (A) Time points including SIV infection, tissue and blood sample collection, and necropsy time points (3 animals/time point). (B) Blood plasma SIV loads (expressed as RNA copies per milliliter) from each animal. Lines connecting data points reflect longitudinal monitoring of individual animals. (Modified from reference 20.) (C) SIV *gag* RNA is not detectable (ND) at 5 dpi, though detectable at 10 dpi and thereafter in all brain regions. Each dot represents the average for 3 animals/time point (9 brain regions per animal). Values are means ± standard errors of the means. (D) SIV *gag* RNA expression is higher in the brainstem and basal ganglia than in the neocortex and cerebellum. Statistical analysis was done by two-way ANOVA using repeated measures and Tukey’s multiple comparisons (**, *P* < 0.01; ***, *P* < 0.001; ****, *P* < 0.0001). Each dot represents the average for 3 animals/time point (brainstem: medulla, pons, and midbrain; neocortex: parietal cortex, frontal cortex, prefrontal cortex, and deep frontal lobe). Values are means ± standard errors of the means.

**FIG 2:**
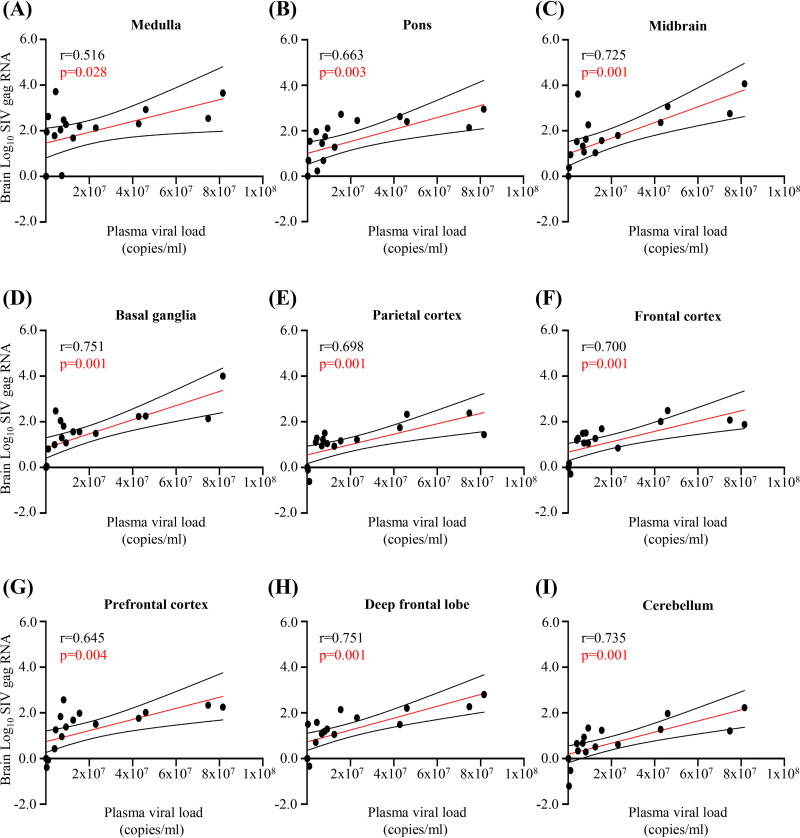
Brain and plasma SIV RNA expression associate significantly in each brain region. (A to I) Correlation analysis shows a strong positive association between plasma viral loads (expressed in RNA copies per milliliter) and brain SIV *gag* RNA levels (normalized to GAPDH levels) in nine distinct brain regions. Each dot represents the mean expression for each brain region in each animal (18 animals). Statistical analysis was done using Pearson’s correlation.

Macaques were monitored for plasma viral loads, and sustained infection was confirmed until the corresponding end points in the parent study (20), which included necropsies at 5, 10, 13, 20, 41, and 90 dpi (Fig. 1A). Plasma SIV RNA was detected from 5 dpi onward through 90 dpi, with peak viremia at 10 dpi (Fig. 1B) (20). Similarly, SIV RNA expression was detected in all brain regions sampled (the brainstem [medulla, midbrain, pons], basal ganglia, neocortex [parietal cortex, frontal cortex, prefrontal cortex, deep frontal lobe], and cerebellum) from 10 dpi onward, but not at 5 dpi (Fig. 1C and D). The highest mean SIV RNA expression was observed in brainstem regions, followed by the basal ganglia, neocortex, and cerebellum, respectively (Fig. 1D). Brain and plasma SIV RNA expression associated significantly in each of the nine brain regions (Fig. 2).

### SIV infection associates with interferon responses throughout the brainstem, basal ganglia, neocortex, and cerebellum, with the highest responses in the brainstem.

**FIG 3:**
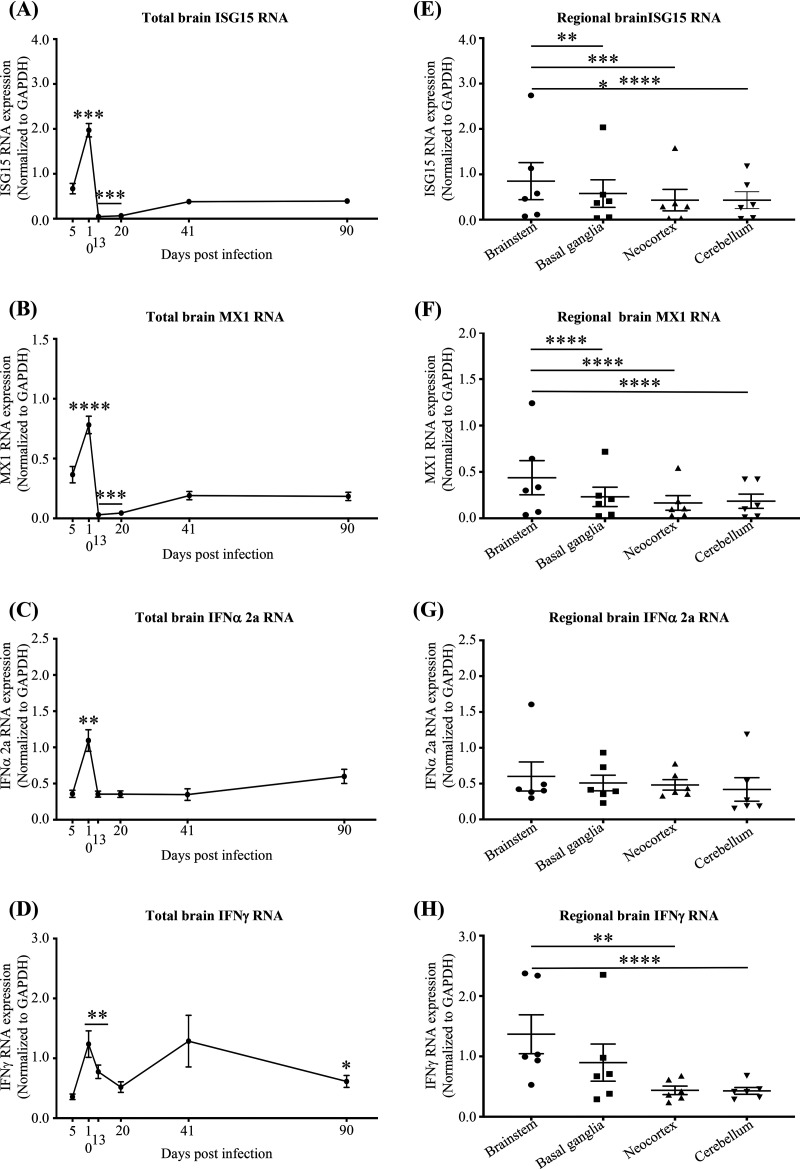
SIV infection associates with higher interferon responses in the brainstem than in other regions. (A to D) RNA expression of IFN response genes ISG15 (A) and MX1 (B), IFN type I (IFN-α 2a) (C), and IFN type II (IFN-γ) (D) over the course of the infection in total brain. SIV infection induces IFN responses. Statistical analysis was done by two-way ANOVA using repeated measures and Tukey’s multiple comparisons (with significance indicated in comparison to 5 dpi [*, *P* < 0.05; **, *P* < 0.01; ***, *P* < 0.001; ****, *P* < 0.0001]). Each dot represents the average for 3 animals/time point (9 brain regions per animal). (E to H) ISG15, MX1, IFN-α 2a, and IFN-γ RNA expression in each region analyzed throughout the infection, respectively. ISG15, MX1, and IFN-γ RNA expression is higher in the brainstem than in other regions. Statistical analysis was done by two-way ANOVA using repeated measures and Tukey’s multiple comparisons (*, *P* < 0.05; **, *P* < 0.01; ***, *P* < 0.001; ****, *P* < 0.0001). Each dot represents the average for 3 animals/time point (brainstem: medulla, pons, and midbrain; neocortex: parietal cortex, frontal cortex, prefrontal cortex, and deep frontal lobe). Values are means ± standard errors of the means.

**FIG 4:**
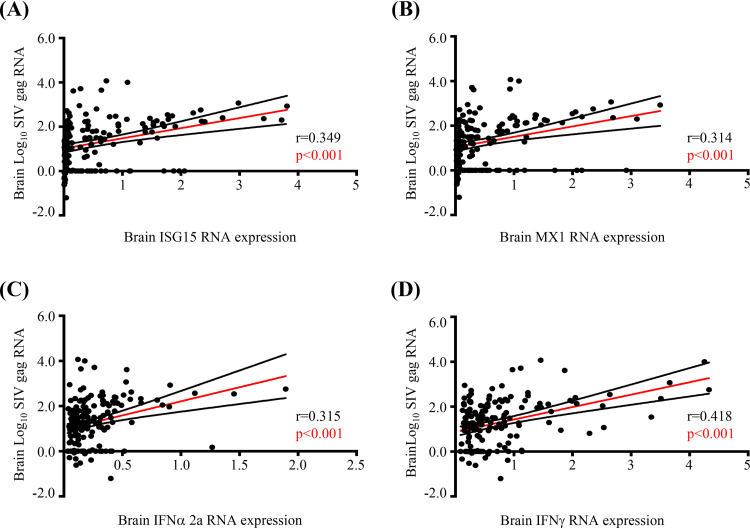
SIV RNA associates positively with IFN responses. (A to D) Correlation analysis shows a strong positive association between IFN response genes (ISG15 and MX1), IFN types I and II (IFN-α 2a and IFN-γ, respectively) and brain SIV *gag* RNA expression (normalized to GAPDH expression). Each dot represents the value for 1 of the 9 regions in an animal (18 animals). Statistical analysis was done using Pearson’s correlation.

Our previous studies of autopsied brain tissue from PLWH and uninfected individuals demonstrated a strong association between type I and II IFN responses, antioxidant responses, and neurocognitive impairment, suggesting cause-effect relationships in chronic HIV infection (18). Therefore, we sought to determine the effects of acute SIV infection on IFN, antioxidant, and neuronal injury responses. We first quantified the RNA expression of IFN response genes (ISG15, MX1), type I and type II IFNs (IFN-α 2a and IFN-γ, respectively), and the astrocyte marker GFAP in each brain region, as markers of neuroinflammation. Time-dependent expression of IFN responses was similar across all brain regions, with a peak at 10 dpi and lower levels thereafter (Fig. 3A to D). Furthermore, although IFN-α 2a did not differ among the regions, expression of ISG15, MX1, and IFN-γ was highest in the brainstem, followed by the basal ganglia, neocortex, and cerebellum, respectively, a pattern similar to that of regional SIV RNA expression (Fig. 3E to H). As expected, brain SIV RNA associated significantly with IFN responses across all brain regions (Fig. 4A to D).

**FIG 5:**
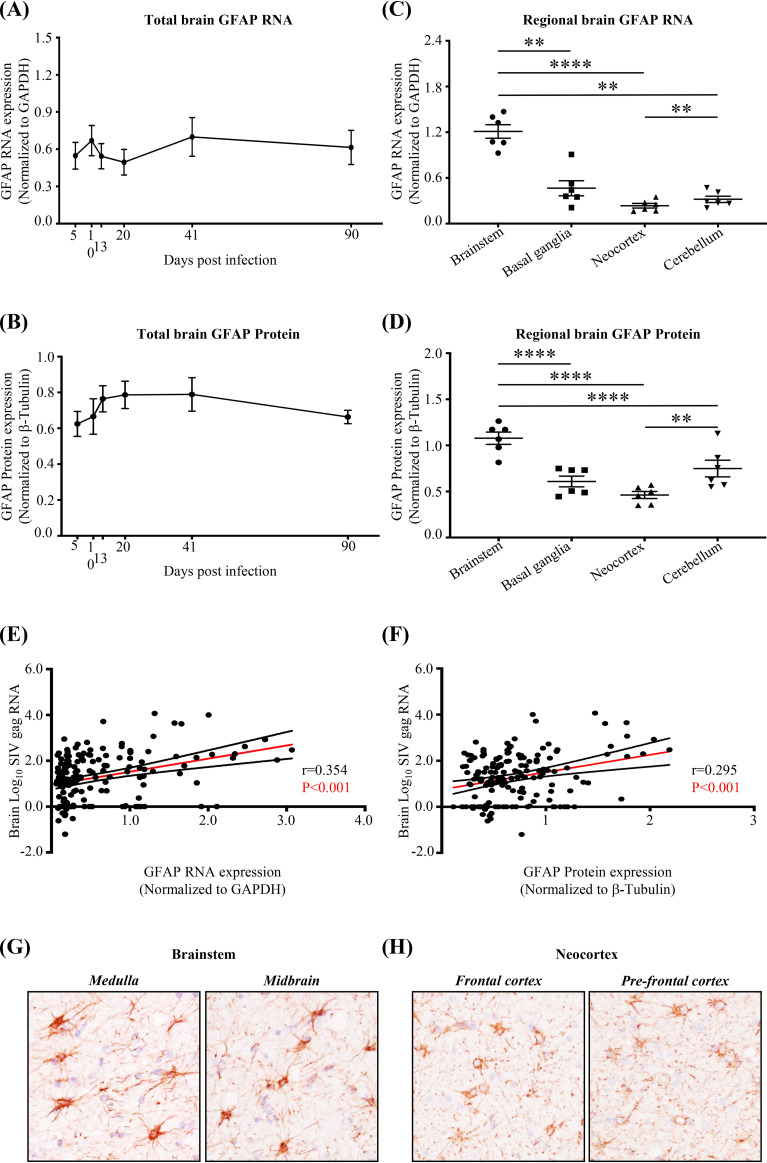
Astrocyte activation (GFAP expression) is higher in the brainstem than in other regions. (A and B) GFAP expression (RNA and protein, respectively) does not change significantly during the course of the infection. Each dot represents the average for 3 animals/time point (9 brain regions per animal). (C and D) GFAP expression (RNA and protein, respectively) is higher in the brainstem than in the basal ganglia, neocortex, and cerebellum. Statistical analysis was done by two-way ANOVA using repeated measures and Tukey’s multiple comparisons (*, *P* < 0.05; **, *P* < 0.01; ***, *P* < 0.001; ****, *P* < 0.0001). Each dot represents the average for 3 animals/time point (brainstem: medulla, pons, and midbrain; neocortex: parietal cortex, frontal cortex, prefrontal cortex, and deep frontal lobe). Values are means ± standard errors of the means. (E and F) Pearson’s correlation analysis shows a positive association between SIV *gag* RNA expression and GFAP expression in the brain. Each dot represents the value for 1 of the 9 regions in an animal (18 animals). (G and H) Representative images from immunohistological detection of GFAP in the brainstem (medulla, midbrain) and neocortex (frontal cortex and prefrontal cortex) from 5 dpi. All images are from the same animal.

Although GFAP expression did not change significantly during the course of infection (Fig. 5A and B), expression was highest in the brainstem (Fig. 5C and D) and associated with SIV RNA expression (Fig. 5E and F). Immunohistochemical staining confirmed higher expression of GFAP in the brainstem than in the neocortex at 5 dpi (Fig. 5G and H).

### SIV infection associates with acute synaptic injury throughout the brainstem, basal ganglia, neocortex, and cerebellum, with spontaneous recovery in all areas except the brainstem.

Previous studies have demonstrated SIV-associated morphological disruption in frontal cortex neurons labeled with synaptophysin (a presynaptic marker) within 12 to 14 dpi without any reduction in microtubule-associated protein-2 (MAP-2, a dendritic marker) or calbindin (dendrites and cell bodies of gamma-aminobutyric acid [GABA]-ergic neurons) expression or any reduction in total neuronal cell counts (1, 9). These observations are consistent with acute synaptic injury without neuronal cell death. Furthermore, in those animals, brain magnetic resonance spectroscopy (MRS) revealed a reduction in the frontal cortex *N*-acetylaspartate (NAA)/creatinine (Cr) ratio, which also indicates neuronal injury (or loss). A similar MRS study of acute SIV infection demonstrated recovery of frontal cortex NAA/Cr ratios by 27 dpi (7), consistent with recovery from injury.

**FIG 6:**
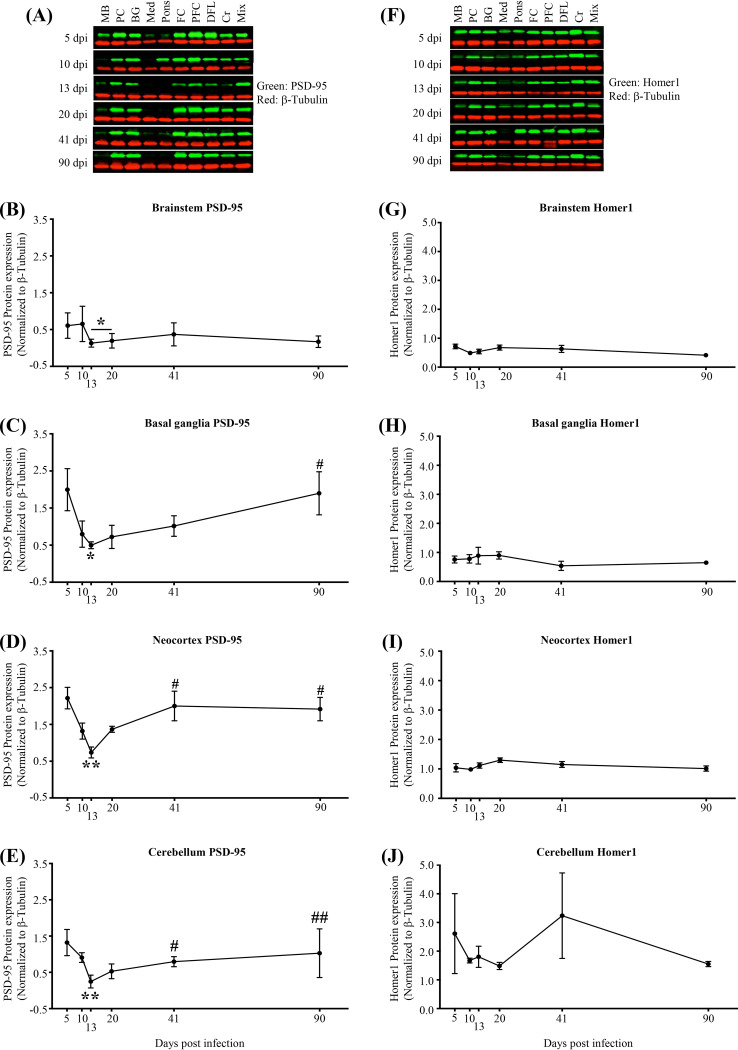
Postsynaptic injury (PSD-95 loss) is detected throughout the brain by 13 dpi, with spontaneous recovery in the neocortex, basal ganglia, and cerebellum but not in the brainstem. (A) Representative immunoblots of PSD-95. MB, midbrain; PC, parietal cortex; BG, basal ganglia; Med, medulla; FC, frontal cortex; PFC, prefrontal cortex; DFL, deep frontal lobe; Cr, cerebellum; Mix, sample made by mixing equal volumes of all 90-dpi samples. Mix was used as a control and was run in all membranes. Each blot was normalized to that sample in each membrane. β-Tubulin was used as a loading control in all membranes. (B to E) PSD-95 expression is reduced in the brainstem, basal ganglia, neocortex, and cerebellum, respectively, by 13 dpi from that at 5 dpi. (B) Brainstem PSD-95 expression (*, *P* < 0.05 [determined by two-way ANOVA with repeated measures and Tukey’s multiple comparisons]). (C) Basal ganglia PSD-95 reduction (*, *P* < 0.05 [determined by one-way ANOVA with Dunnett’s multiple comparisons]) and recovery (#, *P* < 0.05 [determined by one-way ANOVA with a test for linear trend from 13 dpi to 41 and 90 dpi]). (D) Neocortex PSD-95 reduction (**, *P* < 0.01 [determined by two-way ANOVA with repeated measures and Tukey’s multiple comparisons]) and recovery (#, *P* < 0.05 [determined by one-way ANOVA with a test for linear trend from 13 dpi to 41 and 90 dpi]). (E) Cerebellum PSD-95 reduction (**, *P* < 0.01 [determined by one-way ANOVA with Dunnett’s multiple comparisons]) and recovery (#, *P* < 0.05; ##, *P* < 0.01 [determined by one-way ANOVA with a test for linear trend from 13 dpi to 41 and 90 dpi, respectively]). (F) Representative immunoblots of postsynaptic protein Homer1. (G to J) Unchanged Homer1 expression during the course of the infection. Each dot represents the average for 3 animals/time point (brainstem: medulla, pons, and midbrain; neocortex: parietal cortex, frontal cortex, prefrontal cortex, and deep frontal lobe). Values are means ± standard errors of the means.

**FIG 7:**
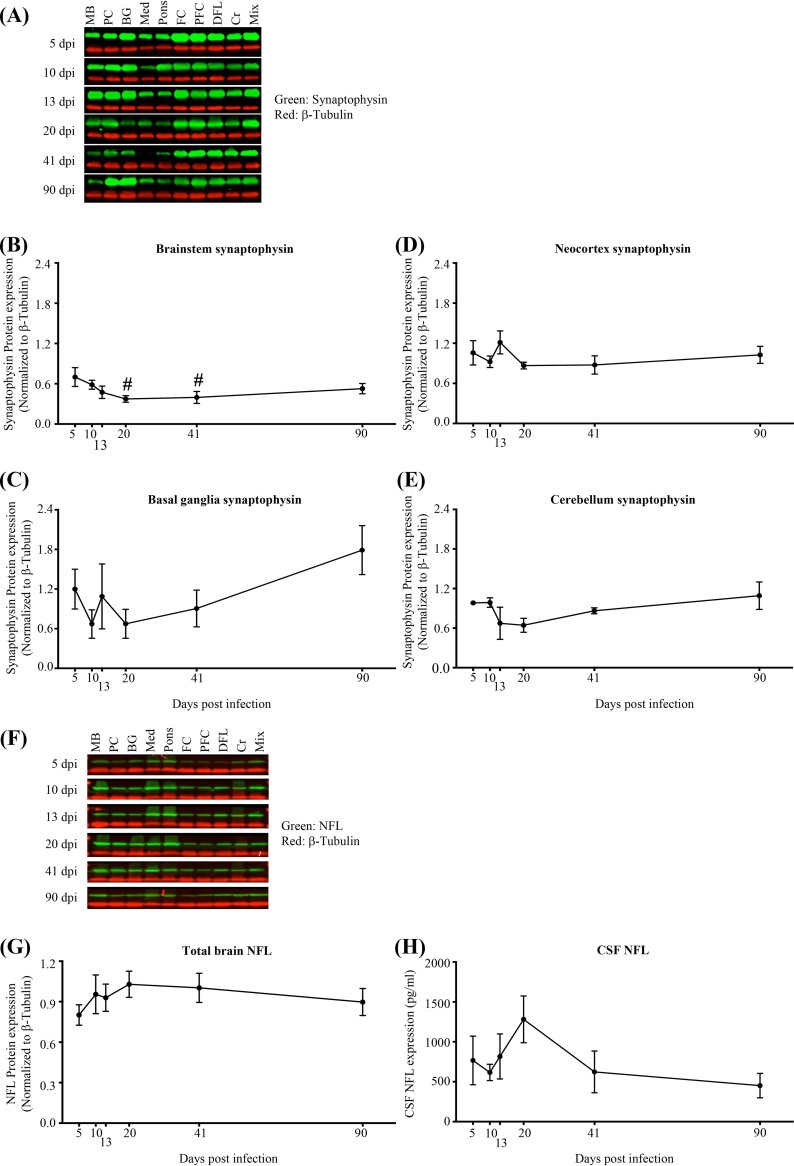
Presynaptic injury (synaptophysin loss) is detected in the brainstem but not in other regions, and no axonal injury (as would be evidenced by NFL loss in brain tissue in any region or a significant increase in CSF NFL) is detected. (A) Representative immunoblots of synaptophysin. MB, midbrain; PC, parietal cortex; BG, basal ganglia; Med, medulla; FC, frontal cortex; PFC, prefrontal cortex; DFL, deep frontal lobe; Cr, cerebellum; Mix, sample made by mixing equal volumes of all 90-dpi samples. Mix was used as a control and was run in all membranes. Each blot was normalized to that sample in each membrane. β-Tubulin was used as a loading control in all membranes. (B) Progressive reduction of synaptophysin in the brainstem (#, *P* < 0.05 [determined by one-way ANOVA with a test for linear trend from 5 dpi to 20 and 41 dpi]). (C to E) Unchanged synaptophysin expression in other regions (*P* > 0.05 [determined by two-way ANOVA with repeated measures and Tukey’s multiple comparisons]). Each dot represents the average for 3 animals/time point (brainstem: medulla, pons, and midbrain; neocortex: parietal cortex, frontal cortex, prefrontal cortex, and deep frontal lobe). Values are means ± standard errors of the means. (F) Representative immunoblots of NFL. (G) Unchanged total-brain NFL expression. Each dot represents the average for 3 animals/time point (9 regions per animal). (H) Unchanged CSF NFL expression. Each dot represents the average for 3 animals/time point. Values are means ± standard errors of the means.

**FIG 8:**
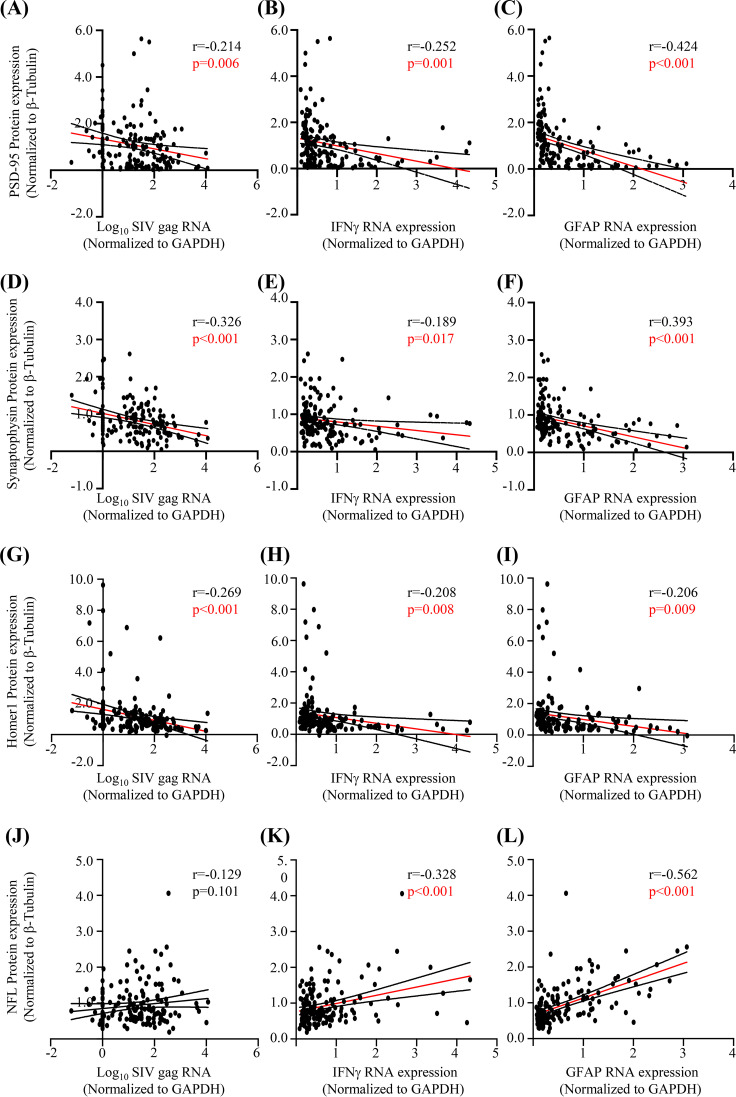
Expression of synaptic proteins associates negatively with SIV RNA and markers of neuroinflammation, and expression of the axonal protein NFL associates positively with neuroinflammation markers. (A to I) Expression of PSD-95 (A to C), synaptophysin (D to F), and Homer1 (G to I) associates negatively with SIV, IFN-γ, and GFAP RNA expression. (J) NFL expression does not associate with SIV RNA. (K and L) NFL expression associates positively with IFN-γ and GFAP RNA expression. Statistical analysis was done using Pearson’s correlation. Each dot represents the value for 1 of the 9 regions in an animal (18 animals).

To more fully assess neuronal injury and potential recovery in response to SIV infection across the brain, we quantified the expression of postsynaptic (PSD-95, Homer1) (Fig. 6), presynaptic (synaptophysin), and axonal (NFL) proteins as markers of neuronal integrity (21) (Fig. 7). Within all regional groups (brainstem, basal ganglia, neocortex, and cerebellum), a significant reduction of PSD-95 expression (postsynaptic *N*-methyl-d-aspartate [NMDA] glutamate receptor linker [22]) by 13 dpi was observed, with recovery of expression by 41 or 90 dpi in all regions except the brainstem (Fig. 6A to E). In contrast, expression of Homer1 (postsynaptic metabotropic glutamate receptor linker [23]) did not change significantly (Fig. 6F to J). Synaptophysin (presynaptic vesicle marker [24]) expression was significantly reduced (*P*, <0.05 by one-way ANOVA and a test for linear trend) from 5 dpi to 20 to 41 dpi in the brainstem but not in the basal ganglia, neocortex, or cerebellum (Fig. 7A to E). Additionally, expression of NFL across all regions or in cerebrospinal fluid (CSF) did not change significantly, although a transient, statistically nonsignificant increase in CSF NFL was observed at 20 dpi (Fig. 7F to H). Furthermore, expression of the synaptic proteins (PSD-95, Homer1, synaptophysin) associated negatively, though weakly, with expression of SIV, IFN-γ, and GFAP RNA (Fig. 8A to I). Finally, while expression of NFL did not associate with SIV RNA (Fig. 8J), it associated positively with IFN-γ and GFAP RNA (Fig. 8K and L).

To address the discordant recovery of PSD-95 expression in the basal ganglia, neocortex, and cerebellum versus the failure of recovery in the brainstem, we focused on potential variations in host neuroprotective antioxidant responses in these regions.

### Brainstem expression of HO-2 decreases in SIV infection concurrently with failure of recovery from synaptic injury.

Previous studies of host protective responses to neuronal injury have demonstrated a cytoprotective role for Nrf2/ARE (antioxidant response element)-driven genes (25). Among these inducible response genes are NQO1, GPX1, Prdx1, and HO-1. The constitutively expressed isoform of HO, HO-2, expresses the same enzymatic function, but it is not Nrf2 inducible. Among the inducible response genes, NQO1 is the most specifically dependent on Nrf2 for upregulation, while the others have additional recognized regulators.

**FIG 9:**
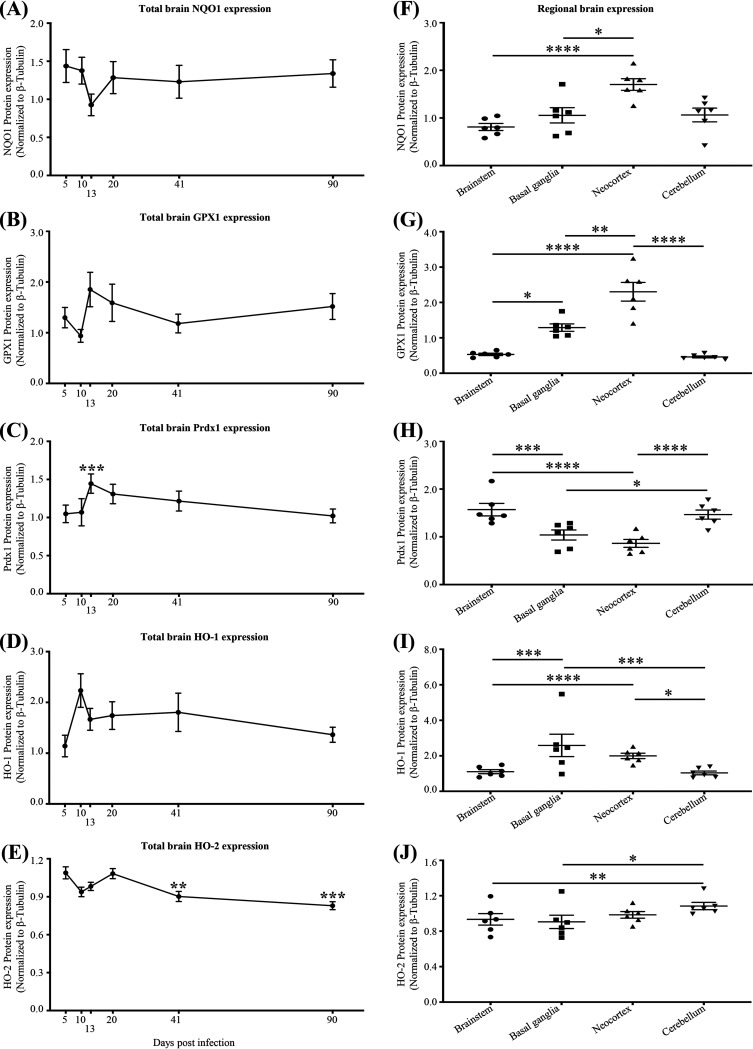
SIV infection associates with progressively reduced expression of HO-2 but not of other antioxidant enzymes in the brainstem. (A to E) Expression of HO-2, but not the antioxidant enzyme NQO1, GPX1, Prdx1, or HO-1, is reduced by 41 dpi and thereafter. Prdx1 is transiently increased at 13 dpi. Asterisks indicate significant differences from 5 dpi (**, *P* < 0.01; ***, *P* < 0.001) by two-way ANOVA with repeated measures and Tukey’s multiple comparisons. Each dot represents the average for 3 animals/time point (9 brain regions per animal). (F to J) Expression of NQO1, GPX1, and HO-1 is lower in the brainstem than in the neocortex, while similar expression levels are observed in the brainstem and cerebellum. HO-2 expression is similar in the neocortex and cerebellum and higher in the cerebellum than in the brainstem and basal ganglia. Asterisks indicate significant differences (*, *P* < 0.05; **, *P* < 0.01; ***, *P* < 0.001; ****, *P* < 0.0001) by two-way ANOVA with repeated measures and Tukey’s multiple comparisons. Each dot represents the average for 3 animals/time point (brainstem: medulla, pons, and midbrain; neocortex: parietal cortex, frontal cortex, prefrontal cortex, and deep frontal lobe). Values are means ± standard errors of the means.

We quantified the protein expression of NQO1, GPX1, Prdx1, HO-1, and HO-2 during the course of infection (Fig. 9A to E). When averaged across all brain regions, expression of HO-2 and Prdx1 changed significantly during the course of infection. HO-2 showed a significant and progressive total-brain reduction (Fig. 9E), while Prdx1 showed a transient increase (at 10 dpi) (Fig. 9C). When regional expression patterns were analyzed, distinct differences were observed. Expression levels of NQO1, GPX1, and HO-1 were significantly lower in the brainstem than in the basal ganglia and neocortex (Fig. 9F, G, and I). HO-2 expression was higher in the cerebellum than in the brainstem and basal ganglia, and was equally low in the brainstem, basal ganglia, and neocortex (Fig. 9J). In contrast, Prdx1 expression was significantly higher in the brainstem than in the basal ganglia and neocortex (Fig. 9H). Similar expression patterns were observed in the brainstem and cerebellum for NQO1, GPX1, Prdx1, and HO-1 (Fig. 9F to I). Among the five enzymes, only GPX1 showed a significant, albeit weak, association (negative) with SIV RNA (data not shown).

The progressive reduction of total-brain HO-2 expression over time (Fig. 9E) occurred concurrently with failure to recover from synaptic injury. Given the changes in HO-2 expression, as well as our previous human brain studies that correlated reduced HO-1 expression with brain HIV load, neuroimmune activation, and cognitive impairment (18), we focused further on regional HO-1 and HO-2 expression.

**FIG 10:**
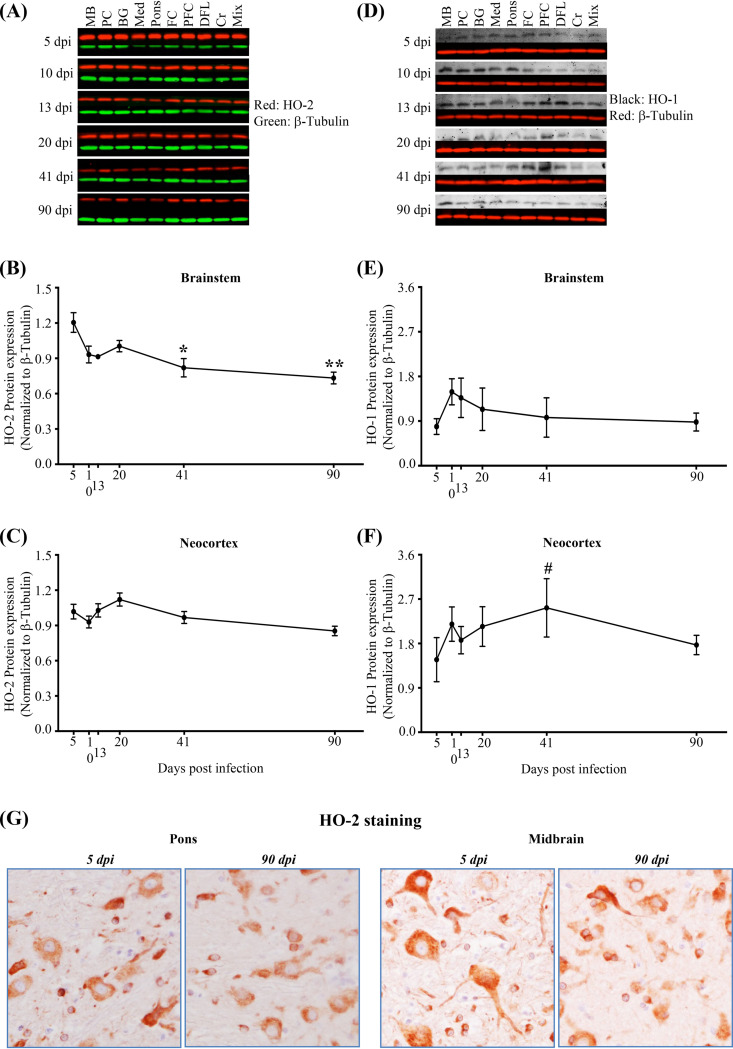
SIV infection associates with progressively reduced expression of HO-2 in the brainstem and progressively increased expression of HO-1 in the neocortex. (A) Representative immunoblots of HO-2. MB, midbrain; PC, parietal cortex; BG, basal ganglia; Med, medulla; FC, frontal cortex; PFC, prefrontal cortex; DFL, deep frontal lobe; Cr, cerebellum; Mix, sample made by mixing equal volumes of all 90-dpi samples. Mix was used as a control and was run in all membranes. Each blot was normalized to that sample in each membrane. β-Tubulin was used as a loading control. (B and C) HO-2 expression is progressively reduced in the brainstem by 41 dpi and thereafter (B), with no change in the neocortex (C). Statistical analysis was done by two-way ANOVA using repeated measures and Tukey’s multiple comparisons; asterisks indicate significant differences (*, *P* < 0.05; **, *P* < 0.01) from 5 dpi. (D) Representative immunoblots of HO-1 with β-tubulin as a loading control. (E and F) HO-1 expression is unchanged in the brainstem (E), while in the neocortex, it is increased during infection (F). Statistical analysis was done using one-way ANOVA with a test for linear trend from 5 to 41 dpi (#, *P* < 0.05). Each dot represents the average for 3 animals/time point (brainstem: medulla, pons, and midbrain; neocortex: parietal cortex, frontal cortex, prefrontal cortex, and deep frontal lobe). Values are means ± standard errors of the means. (G) Representative immunohistochemical staining for HO-2 expression in the brainstem (pons, midbrain) at 5 and 90 dpi. Images taken at the same time point represent two brainstem subregions from the same animal.

We observed distinct time-dependent differences in HO-1 and HO-2 expression in the brainstem and neocortex during the course of infection (Fig. 10). With a deeper analysis of regional HO expression, we found that the progressive reduction of total-brain HO-2 expression (Fig. 9E) was driven by its reduction in the brainstem (Fig. 10A to C). Within the brainstem, expression of HO-2 was significantly reduced at 41 and 90 dpi. Brainstem expression of HO-1 did not change (Fig. 10D and E), but HO-1 expression increased significantly within the neocortex (by a test for linear trend from 5 to 41 dpi) (Fig. 10F). Analysis of HO-2 expression in the brainstem by immunohistochemistry (IHC) supports the evidence for reduced HO-2 expression in the brainstem from 5 to 90 dpi (Fig. 10G).

## DISCUSSION

We present evidence for acute SIV-induced synaptic injury globally within the brain (brainstem, basal ganglia, neocortex, and cerebellum) in immunocompetent rhesus macaques, with spontaneous recovery in all regions except the brainstem. The pattern of recovery from injury and the contrasting levels of HO-2 isoform expression in distinct areas that recover or fail to recover are consistent with a role for HO-2 in recovery from acute SIV-induced synaptic injury. The early regional synaptic injury associated concurrently with regional expression of SIV RNA, IFN responses, and GFAP (26–33). The negative associations between SIV RNA and synaptic markers and between neuroinflammatory markers and synaptic markers, and the positive association between SIV RNA and neuroinflammatory markers, are consistent with SIV driving synaptic injury through induction of neuroinflammation. Throughout infection, inflammatory responses and SIV RNA expression were consistently higher in the brainstem than in the basal ganglia, neocortex, and cerebellum, likely promoting ongoing injury. Among the antioxidant enzymes, HO-2 was expressed at progressively lower levels in the brainstem, which failed to recover from synaptic injury.

We speculate that progressive reduction of brainstem HO-2 expression during acute infection may contribute to the failure of recovery from acute SIV-induced synaptic injury. In addition, we recognize that progressively increasing HO-1 expression in the neocortex (5 to 41 dpi) may contribute to recovery from acute synaptic injury in those regions. In support of this, we showed that SIV infection associates with the following: (i) higher SIV RNA levels in the brainstem than in other regions; (ii) consistent associations between SIV RNA and inflammation in all brain regions; (iii) early synaptic injury marked by reduced PSD-95 expression at 13 dpi in the brainstem, basal ganglia, neocortex, and cerebellum and progressively reduced synaptophysin in the brainstem; (iv) progressively reduced HO-2 expression in the brainstem but not in other regions; (v) increasing PSD-95 in the basal ganglia, neocortex, and cerebellum, but not in the brainstem, after acute injury; and (vi) progressively increasing HO-1 expression in the neocortex during recovery from acute synaptic injury. Notably, we observed no change in NFL expression across all brain regions and a transient, albeit statistically nonsignificant, elevation of CSF NFL during the course of infection. Because NFL is a major component of axons (34), our results are consistent with the hypothesis that little, if any, permanent axonal injury occurs during acute SIV infection despite the presence of synaptic injury.

Apparent spontaneous recovery from acute SIV neuronal injury in the frontal cortex has been suggested previously (7), and our study extends the analysis of brain injury and recovery during acute SIV infection by distinguishing regional brain responses and by demonstrating concurrent changes in host antioxidant enzyme expression. Previous immunohistological studies demonstrated SIV-associated morphological disruption in neurons in the frontal cortex labeled with synaptophysin within 12 to 14 dpi, without a reduction in microtubule-associated protein-2 (MAP-2, a dendritic marker), calbindin (GABA-ergic neurons), or neuronal cell counts (1, 9). These observations are consistent with acute synaptic injury in the frontal cortex without loss of neurons. Evidence for recovery from such acute SIV injury has been provided by MRS studies demonstrating recovery of frontal cortex NAA/Cr ratios within several weeks of acute SIV infection (7, 8).

Nonetheless, apparently irreversible acute neuronal injury may also occur within the frontal cortex in SIV infection. An early account of SIV infection of rhesus macaques with the molecularly cloned SIV strain SIV_mac239_ (T lymphocyte-tropic), derived from the SIV_mac251_ swarm (T lymphocyte- and macrophage-tropic) (6, 35), revealed that invasion of the meninges and brain parenchyma elevated CSF IgG indices and levels of quinolinic acid, indicating acute intrathecal immune activation and neurotoxin production (36). Another rhesus study of acute SIV_mac239_ infection used a combination of brain MRS and postmortem brain analyses to demonstrate frontal cortex neuronal injury (reduced expression of synaptophysin, NAA, and calbindin, and astrocyte activation [increased GFAP]) (1).

Our findings are consistent with the hypothesis that, in the early stages (days) of SIV infection, synaptic injury may predominate, with little, if any, axonal injury or neuronal death. Two of three synaptic markers, PSD-95 and synaptophysin, but not Homer1, were reduced during acute infection and beyond, while no change in brain NFL was observed, and a transient, statistically nonsignificant rise in CSF NFL was observed. In studies of PLWH, significant elevations of NFL in plasma and CSF associate with severe neurocognitive impairment associated with chronic HIV infection, but such elevations are not consistently seen in acute HIV infection (37). In contrast to axonal NFL expression, PSD-95 expression is limited to the postsynaptic neuronal membrane of excitatory synapses, where it associates with *N*-methyl-d-aspartate (NMDA) receptors (38, 39), and synaptophysin is localized to presynaptic vesicles at axodendritic or axosomatic synapses in mature neurons of multiple types (40). Homer1 localizes with postsynaptic type 1 metabotropic glutamate receptors and promotes association with NMDA receptors (41). In contrast to the slow turnover rate of PSD-95, certain Homer1 isoforms exhibit rapid turnover and redistribution rates in response to neuronal activation (42). The decrease in PSD-95 and synaptophysin and the lack of change in NFL throughout the brain (and in CSF) are most consistent with synaptic and not axonal injury. Because the anti-Homer1 antibody used for Western blotting does not distinguish among the several known Homer1 isoforms (Table 1), we speculate that discordant changes in Homer 1 isoforms in response to neuronal activity and/or injury (43, 44) may be undetected.

**TABLE 1:** Information on all antibodies used in Western blotting and IHC

Antibody	Host	Isotype	Size (kDa)	Dilution^*a*^	Catalog no.	Company
Anti-HO-1	Rabbit	Polyclonal	32	1:500	SPA-894	Enzo Life Sciences
Anti-HO-2	Mouse	Monoclonal IgG2a	36	1:1,000 (WB); 1:500 (IHC)	MA5-25749	Invitrogen
Anti-Prdx1	Rabbit	Monoclonal	22	1:1,000	8732	Cell Signaling Technologies
Anti-NQO1	Mouse	Monoclonal IgG1	33	1:5,000	ab28947	Abcam
Anti-GPX1	Rabbit	Monoclonal	33	1:5,000	3286S	Cell Signaling Technologies
Anti-GFAP	Mouse	Monoclonal IgG1	48	1:2,000 (WB); 1:1,000 (IHC)	3670S	Cell Signaling Technologies
Anti-PSD-95	Mouse	Monoclonal IgG2a	95	1:1,000	MAB1596	Millipore
Anti-Homer1	Rabbit	Monoclonal	46	1:4,000	ab184955	Abcam
Anti-NFL	Rabbit	Monoclonal	66	1:10,000	ab52989	Abcam
Anti-synaptophysin	Mouse	MonoclonalIgG1	37	1:1,000	ab8049	Abcam
Anti-β-tubulin	Mouse	Monoclonal IgG2b	50	1:10,000	86298S	Cell Signaling Technologies
Anti-β-tubulin	Rabbit	Monoclonal	50	1:3,000	2128S	Cell Signaling Technologies
IgG1	Mouse	Isotype control		Same as GFAP	ab91353	Abcam
IgG2a	Mouse	Isotype control		Same as HO-2	ab18413	Abcam

a WB, Western blotting.

Why our studies revealed evidence for acute synaptic injury without clear evidence for axonal injury is uncertain; however, selective vulnerability of synapses to metabolic stress in neurodegenerative diseases is well known (45–47). Within synapses, abundant ATP is generated and consumed, leading to the production of free radicals and possibly oxidative-stress-associated injury. One might therefore expect that antioxidant enzymes would be important in maintaining healthy synapses. Because HO-2 predominates in neurons while HO-1 predominates in glia and other nonneuronal cells, we speculate that the partial loss of HO-2 expression within the brainstem contributes directly to the persistence of synaptic injury in the brainstem. Several studies demonstrate that HO-2 deficiency (HO-2 knockout mice) associates with poor recovery from traumatic brain injury (48), which may be linked to increased lipid peroxidation-associated neuronal loss after such injury (14). Although HO-1 is rapidly induced and HO-2 is primarily constitutively expressed and only slowly inducible (49, 50), each catalyzes the same enzymatic degradation of free heme, a potent intra- and extracellular prooxidant, to the enzymatic products carbon monoxide and biliverdin, each of which has anti-inflammatory, neuroprotective, and regenerative effects within the CNS (51, 52).

We propose a role for HO-2 in modulating recovery from SIV synaptic injury, based on our data demonstrating a progressive reduction in HO-2, concurrent with persistent reduction of PSD-95 and progressive reduction of synaptophysin in the brainstem, but not in other brain areas, throughout SIV infection. Reduced HO-2 expression associates with inflammation- and ischemia-induced neuronal injury (13, 53–56). However, what accounts for the progressive reduction of HO-2 in the brainstem in SIV infection is unclear. In various model systems, induced ischemia can reduce HO-2 expression (49). Notably, the HO-2 3′ untranslated region (3′ UTR) mRNA has an oxygen-sensing consensus sequence that regulates transcriptional responses to oxygen variations (57–59). It is possible that reduced brainstem blood flow during acute SIV infection could be a factor contributing to reduced HO-2 expression, although this is unexplored. Although chronic HIV and SIV infections are associated with reduced cerebral blood flow (CBF) in cortical and subcortical regions (60, 61), little is known about CBF in acute HIV infection. In rhesus macaques, acute SIV infection is associated with reduced CBF in the caudate, parietal cortex, and prefrontal cortex within 4 to 8 weeks (62).

**FIG 11:**
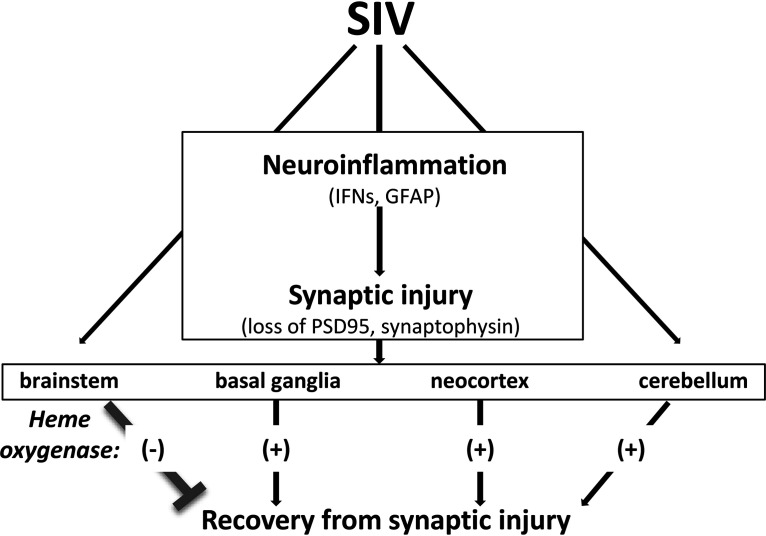
Proposed role for heme oxygenase loss in persistent synaptic injury in SIV-infected macaques. Acute SIV infection induces neuroinflammation, which leads to global brain synaptic injury, indicated by loss of PSD-95 and synaptophysin. Under these conditions, recovery is achieved in brain regions with stable expression of heme oxygenase (basal ganglia, neocortex, cerebellum), while failure to recover (brainstem) is associated with progressive loss of heme oxygenase (isoform HO-2).

In summary, our results show that progressive reduction of HO-2 expression in the brainstem during acute SIV infection is associated with neuroinflammation, SIV load, and acute synaptic injury. This HO-2 reduction, which is not observed in other brain areas, may contribute to the failure of the brainstem to recover from such injury (Table 2). In other brain regions, the maintenance of stable HO-2 levels during infection may support recovery from such acute synaptic injury (Table 3). A role, if any, for HO-1 in supporting recovery is less clear, although its expression is increased in the neocortex during recovery. Induction of HO expression may provide neuroprotection and enhanced neuronal recovery from SIV- and HIV-induced injury (see the diagram in Fig. 11).

**TABLE 2:** Markers in the brainstem, with their functions and progression during infection

Marker	Progression^*a*^	
	From 5 dpi to injury time (acute infection)	From injury time to end of study (90 dpi)
Viral (*gag* RNA)	↑	→
Inflammatory		
ISG15	↑	↓
MX1	↑	↓
IFN-α 2a	↑	↓
IFN-γ	↑	→
GFAP (RNA, astrocytes)	NS	NS
GFAP (protein)	NS	NS
Neuronal		
PSD-95 (postsynaptic protein)	↓	→
Synaptophysin (presynaptic protein)	↓	→
Homer1 (postsynaptic protein)	NS	NS
NFL (axonal protein)	NS	NS
NFL (CSF, soluble NFL)	NS	NS
Antioxidant enzymes		
NQO1	NS	NS
GPX1	NS	NS
Prdx1	↑	↓
HO-1	NS	NS
HO-2	↓	↓

a Arrows indicate increasing (↑), decreasing (↓), or change maintained (→) levels; NS, no significant change.

**TABLE 3:** Markers in the neocortex, with their functions and progression during infection

Marker	Progression^*a*^	
	From 5 dpi to injury time (acute infection)	From injury time to end of study (90 dpi)
Viral (*gag* RNA)	↑	→
Inflammatory		
ISG15	↑	↓
MX1	↑	↓
IFN-α 2a	↑	→
IFN-γ	↑	↓
GFAP (RNA, astrocytes)	NS	NS
GFAP (protein)	NS	NS
Neuronal		
PSD-95 (postsynaptic protein)	↓	↑
Synaptophysin (presynaptic protein)	NS	NS
Homer1 (postsynaptic protein)	NS	NS
NFL (axonal protein)	NS	NS
NFL (CSF, soluble NFL)	NS	NS
Antioxidant enzymes		
NQO1	NS	NS
GPX1	NS	NS
Prdx1	↑	↓
HO-1	↑	→
HO-2	NS	NS

a Arrows indicate increasing (↑), decreasing (↓), or change maintained (→) levels; NS, no significant change.

## MATERIALS AND METHODS

### Ethical statement for use of nonhuman primates.

As reported previously (20), this study was performed following the protocol (number #YER-2002541-121716GA) approved by the Institutional Animal Care and Use Committee (IACUC) of Emory University, in accordance with the Animal Welfare Act and other federal statutes and regulations relating to animals. Animals were housed at the Yerkes National Primate Research Center (YNPRC) at Emory University (Atlanta, GA) following the guidelines established by the National Institutes of Health (NIH), and under the supervision of the AAALAC (Association for the Assessment and Accreditation of Laboratory Animal Care)-accredited Division of Animal Resources.

### Animals.

Eighteen healthy Indian rhesus macaques (32 to 38 months of age; 13 males, 5 females) were used. Animals were necropsied after SIV inoculation at different time points (5, 10, 13, 20, 41, or 90 dpi; 3 animals/time point). All animals were Mamu A*01 (major histocompatibility complex [MHC] class I) positive and Mamu B*08 and Mamu B*17 negative. In accordance with YNPRC IACUC guidelines, animals were quarantined for 30 to 45 days prior to study entry.

### SIV infection and plasma viral loads.

Animals were infected intravenously (i.v.) with SIV_mac251_ at 500 50% tissue culture infective doses (TCID_50_) (10.8 ng/ml p27) (Nancy Miller, NIAID, NIH) as described previously (20). Plasma viral loads were determined by quantitative PCR (qPCR) (sensitivity, 60 copies/ml; Emory University Center for AIDS Research [CFAR] Virology Core).

### Brain and cerebrospinal fluid harvesting.

Brain tissue and cerebrospinal fluid (CSF) were harvested at the necropsy time points. CSF and tissues for Western blotting or RNA analysis were immediately frozen (–80°C). Tissues for immunohistochemistry staining were preserved in paraformaldehyde for paraffin embedding. Samples were collected at the YNPRC and shipped to the University of Pennsylvania. Brain tissue for regional RNA and protein quantification was harvested from contiguous sites within each brain region: the brainstem (medulla, midbrain, pons), basal ganglia, neocortex (parietal cortex, frontal cortex, prefrontal cortex, deep frontal lobe), and cerebellum.

### Brain SIV RNA and biomarker determinations.

Total RNA was prepared with an RNeasy lipid extraction kit (Qiagen) and quantified with a NanoDrop 2000c UV-Vis spectrophotometer (Thermo Fisher Scientific). A 25-μg portion of total RNA was reverse transcribed to cDNA using a High-Capacity RNA-to-cDNA kit (Applied Biosystems). Relative gene expression was determined using 1 ng RNA, TaqMan Fast Universal PCR master mix (Applied Biosystems), and appropriate TaqMan primer/probe sets (Applied Biosystems). RNA expression relative to glyceraldehyde-3-phosphate dehydrogenase (GAPDH) expression was normalized to the same RNA loading control used in all batches assayed.

TaqMan primers and probes used included the following: SIV *gag* forward primer sequence, CAATTTTACCCAGGCATTTAATGTT; SIV *gag* reverse primer sequence, GCAGAGGAGGAAATTACCCAGTAC; SIV *gag* probe, 6-carboxyfluorescein (FAM)-TGTCCACCTGCCATTAAGCCCGA-6-carboxytetramethylrhodamine (TAMRA) (63). The assays used for type I IFN genes were Rh02915441_g1 for ISG15, Rh00895608_m1 for MX1, and Rh04256335_s1 for IFNA2; for the type II gene IFNG, assay Rh02621721_m1 was used. Rh00909240_m1 was used for the astrocyte marker GFAP, and Rh02621745_g1 was used for GAPDH. All inflammatory marker and GAPDH probes were from Thermo Fisher Scientific.

### Western blotting.

Brain tissue lysates were prepared by homogenization (∼100 mg of tissue) by silica bead beating and sonication in 7 volumes of buffer (10 mM Tris-HCl [pH 7.8], 0.5 mM dithiothreitol, 5 mM MgCl_2_, 0.03% Triton X-100) containing phosphatase inhibitor cocktail set II (EMD Millipore) and a protease inhibitor cocktail (Sigma-Aldrich). Protein was quantified using the DC (detergent-compatible) protein assay (Bio-Rad). Equivalent amounts of proteins were added to Laemmli sample buffer (50 mM Tris-HCl [pH 6.8], 2% SDS, 10% glycerol, 12.5 mM EDTA, 0.002% bromophenol blue) with 2.5% 2-mercaptoethanol, and mixtures were denatured at 95°C for 10 min. Proteins were resolved on an SDS-PAGE gel and transferred overnight to poly(vinylidene difluoride) (PVDF) membranes (4°C). Membranes were blocked with Odyssey blocking buffer (phosphate-buffered saline [PBS]) (LI-COR Biosciences) and incubated with primary antibody overnight (4°C). TRDye-conjugated secondary antibodies (LI-COR Biosciences) were used to detect the primary antibody. Background-corrected signal quantification of protein bands was determined using Image Studio Lite software (LI-COR Biosciences). One sample made by mixing equal volumes of all 90-dpi samples (Mix) was used as a control and was run in all membranes. Each blot was normalized to that sample in each membrane. Because we had 18 animals with 9 regions per animal to analyze, and because it was not possible to load all samples and run them in one gel, we decided to prepare a mix control sample (Mix) to run in each Western blot. The use of this control made it possible to compare protein expression among all animals and regions analyzed. There was not a specific reason for choosing samples from the 90-dpi animals to prepare the Mix, since choosing a combination of samples from any day postinfection would be considered similarly rationalized. Information on the antibodies used in this study is shown in Table 1.

### IHC analyses.

One of two adjacent portions of each brain region per animal was fixed by immersion in 4% paraformaldehyde for 48 h, washed in 1× PBS, and transferred to 70% ethanol before paraffin embedding and sectioning (10 μm). The remaining portion was frozen and processed for protein and RNA quantification, as described above. For immunohistochemistry (IHC) staining, sections were deparaffinized in xylene, rehydrated in ethanol and distilled water, quenched with 3% H_2_O_2_ for 30 min, and microwaved 15 min in 10 mM citrate buffer (pH 6) for epitope exposure. Primary antibodies and isotype-matched control antibodies (Table 1) were used at identical concentrations for staining with an overnight incubation (4°C), two successive washes, and incubation with an appropriate biotin-conjugated secondary antibody. Signal was amplified using an avidin-biotin horseradish peroxidase system according to the manufacturer’s instructions (Vectastain ABC kit; Vector Laboratories, Inc.). A chromophore reaction was developed with diaminobenzidine and hydrogen peroxide. Sections were dehydrated and mounted with Cytoseal 60 (Thermo Scientific, Waltham, MA, USA). All sections were viewed using a Nikon Eclipse 80i microscope, and images were acquired using a Nikon DS-Fi2 digital camera (Micro Video Instruments, Avon, MA).

### CSF neurofilament assay.

CSF collected from each animal at the time of necropsy was used to quantify NFL by a singleplex assay (Meso Scale Discovery, Rockville, MD, USA) as described in the R-PLEX protocol.

### Data acquisition and statistical analyses.

Statistical analyses were performed using GraphPad Prism software. Data were analyzed by Student’s *t* test, one-way analysis of variance (ANOVA) with multiple comparisons, one-way ANOVA with a test for linear trend, or two-way ANOVA with repeated measures. Tukey’s and Dunnett’s post hoc tests were used for multiple comparisons. Pearson’s correlation was used for comparisons between two continuous variables.
